# A non-targeted metabolomics study on *Xylella fastidiosa* infected olive plants grown under controlled conditions

**DOI:** 10.1038/s41598-020-80090-x

**Published:** 2021-01-13

**Authors:** Asmae Jlilat, Rosa Ragone, Stefania Gualano, Franco Santoro, Vito Gallo, Leonardo Varvaro, Piero Mastrorilli, Maria Saponari, Franco Nigro, Anna Maria D’Onghia

**Affiliations:** 1 Dipartimento di Scienze Agrarie e Forestali (DAFNE), Università Degli Studi Della Tuscia, Via San Camillo de Lellis, 01100 Viterbo, Italy; 2 Dipartimento di Ingegneria Civile, Ambientale, del Territorio, Edile e di Chimica (DICATECh), Politecnico di Bari, Via Orabona 4, 70125 Bari, Italy; 3 Innovative Solutions S.R.L. – Spin Off del Politecnico Di Bari, Zona H 150/B, 70015 Noci, BA Italy; 4 Centre International de Hautes Etudes Agronomiques Méditerranéennes (CIHEAM) of Bari, Via Ceglie 9, 70010 Valenzano, BA Italy; 5 Istituto Per La Protezione Sostenibile Delle Piante, CNR, SS Bari, Via Amendola 165/A, 70126 Bari, Italy; 6 Dipartimento di Scienze del Suolo, della Pianta e degli Alimenti, Università Degli Studi di Bari ″Aldo Moro″, Via Amendola 165/A, 70126 Bari, Italy

**Keywords:** Biotic, Metabolomics

## Abstract

In the last decade, the bacterial pathogen *Xylella fastidiosa* has devastated olive trees throughout Apulia region (Southern Italy) in the form of the disease called “Olive Quick Decline Syndrome” (OQDS). This study describes changes in the metabolic profile due to the infection by *X. fastidiosa* subsp. *pauca* ST53 in artificially inoculated young olive plants of the susceptible variety *Cellina di Nardò*. The test plants, grown in a thermo-conditioned greenhouse, were also co-inoculated with some xylem-inhabiting fungi known to largely occur in OQDS-affected trees, in order to partially reproduce field conditions in terms of biotic stress. The investigations were performed by combining NMR spectroscopy and MS spectrometry with a non-targeted approach for the analysis of leaf extracts. Statistical analysis revealed that *Xylella*-infected plants were characterized by higher amounts of malic acid, formic acid, mannitol, and sucrose than in *Xylella*-non-infected ones, whereas it revealed slightly lower amounts of oleuropein. Attention was paid to mannitol which may play a central role in sustaining the survival of the olive tree against bacterial infection. This study contributes to describe a set of metabolites playing a possible role as markers in the infections by *X. fastidiosa* in olive.

## Introduction

In recent years, world-renowned Apulian olive groves (southern Italy) have experienced a critical turning point in their history with the appearance of a severe disease that was promptly called Olive Quick Decline Syndrome (OQDS). OQDS is typically characterized by a massive leaf scorching and a scattered desiccation of twigs and branches, which progressively extends to the entire tree canopy until death, particularly in the case of susceptible cultivars^1^.

OQDS is caused by a quarantine bacterial pathogen, *Xylella fastidiosa,* of American origin, belonging to the EPPO A.2 list^2,3^. *X. fastidiosa* is a Gram-negative xylem-limited bacterium that has a wide host range^4^ and causes a number of diseases of economically important crops, such as Pierce’s disease of grapevine, plum leaf scald, phony peach, almond leaf scorch, citrus variegated chlorosis and coffee leaf scorch^5^. *X. fastidiosa* is classified into three formally accepted subspecies, namely *fastidiosa*, *multiplex* and *pauca*^6^. Additionally, several *X. fastidiosa* subspecies have been proposed including *sandyi*^7^, *tashke*^8^ and *morus*^9^. The Apulian strain affecting olive trees belongs to the sequence type 53 within the subspecies *pauca*^10^. *X. fastidiosa* is vectored by xylem sap-feeding insects, belonging to the Auchenorrhyncha suborder^11^. So far, surveys on *X. fastidiosa* transmission in Apulia led to the identification of the widespread meadow spittlebug, *Philaenus spumarius*, as the primary insect vector^12^. Since its first detection in Italy*, X. fastidiosa* is threatening the European and Mediterranean agriculture and landscape. After the first finding of *X. fastidiosa* subspecies *pauca* in Italy, several outbreaks of the bacterium have been reported in Southern Europe: in France with the subspecies *multiplex*, *fastidiosa* and *sandyi* (southern region of Côte d’Azur and island of Corsica); in Spain with the subspecies *multiplex, fastidiosa* and *pauca* (Balearic Islands and province of Alicante); in Portugal with the subspecies *multiplex* (Vila Nova de Gaia); and in the Italian Tuscany region with the subspecies *multiplex*. It is assumed that these *X*. *fastidiosa* introductions originate from the import of *X. fastidiosa*-infected plants^13–15^. *X. fastidiosa* epidemiology and dynamics differ for each pathosystem that is characterised by the bacterium genotypes, host plant species, insect vectors and environmental conditions^16^. Although the pathogenicity of *X. fastidiosa* has been studied in the Americas for several decades, there are no therapeutic solutions to suppress the bacterial development in infected plants^17^. Therefore, the need to perceive the specific host plant-pathogen interactions has driven research towards studies of the molecular and physiological mechanisms generated by *X. fastidiosa* infection. Scientific investigations on *X. fastidiosa* epidemiology in Apulian olive groves showed a diversity in the responses of olive cultivar to the infection. In particular, the *Leccino* variety develops milder symptoms compared to those observed on the *Cellina di Nardò* and *Ogliarola salentina* varieties^18,19^. Correspondingly, the bacterial populations size measured in the infected plants showed a significantly lower titre in *Leccino* compared to the susceptible varieties *Cellina di Nardò* and *Ogliarola salentina*^2,18,19^. In addition, studies were conducted under field conditions to investigate cultivar responses to bacterial infection in terms of metabolic compounds, analysed by Mass Spectrometric (MS) methods. Luvisi et al. assessed phenolic compounds in four olive tree varieties (*Cellina di Nardò, Ogliarola di Lecce*, *Frantoio* and *Leccino*) and the results showed a reduction in hydroxytyrosol glucoside and an increase in quinic acid content in all *X. fastidiosa*-infected trees^20^. Sabella et al. reported that the amount of quinic acid, a precursor of lignin which is known to be important for disease defence and water transport in vascular plants^21, 22^, increased only in infected *Leccino* cultivar^23^. It has also been reported that azelaic acid has been accumulated in *Cellina di Nardò* and *Leccino* olive trees after being infected by *X. fastidiosa*^24^. Another study explored the amounts of volatile compounds in *Cellina di Nardò* and *Ogliarola* olive trees, noting a marked presence of methyl esters in *Xylella-*infected trees^25^. Additionally, it was reported that p-coumaric acid disappeared after the infection of *Cellina di Nardò* and *Leccino* olive trees. Furthermore, the amounts of flavonoids (such as quercetin, kaempferol and genistein) and oleanolic, salicylic and kynurenic acids increased only in infected *Leccino* trees^26^.

Lastly, other studies conducted under field conditions by using the Nuclear Magnetic Resonance (NMR) Spectroscopy technique, characterised the metabolic profile of *X. fastidiosa*-infected *Ogliarola salentina* and *Cellina di Nardò*, before and after applying a fertilizer containing zinc, copper, and citric acid^27,28^. Results of the first work showed that untreated *Cellina di Nardò* trees exhibited a lower polyphenols and a higher sugar content with respect to the fertilized trees; however, untreated *Ogliarola salentina* trees showed a higher content of polyphenol molecules and a lower sugar content with respect to the treated ones^27^. Results of the second work showed that, in both varieties, quinic acid, the aldehydic form of oleuropein, ligstroside and phenolic compounds, were found higher for the untreated olive trees in comparison with the trees treated with fertilizers. Moreover, only *Ogliarola salentina* fertilized trees exhibited an increase in malic acid^28^.

Information on the metabolic profile of infected olive trees can therefore help to understand the chemical changes generated by *X. fastidiosa* colonization. This type of data can be achieved through the metabolomic approach that is defined as "the quantitative measurement of the dynamic multiparametric metabolic response of living systems to pathophysiological stimuli or genetic modification"^29,30^. In other words, the metabolomic approach is a bio-analytical methodology applied to investigate complex metabolic patterns associated with the response of an organism to physiological or pathological events. Metabolomics datasets are commonly acquired by either MS or NMR^31^, that are two complementary powerful tools. Combining MS and NMR datasets greatly promotes the coverage of the metabolome and provids a detailed high-throughput analysis of metabolic changes due to diseases, drug treatments, or other environmental stimuli^32^. Comprehensive analysis of such metabolic profiles adopting chemometric and statistical methods, enables the identification of a particular or a combination of metabolites as reliable bio-markers that are crucial in revealing and following changes in biosystems^30^.

Considering that *X. fastidiosa* infection has been outspreading through the southern Apulia and that all studies related to metabolomic profiling of olive trees were conducted under field conditions, it appears of utmost importance to study the metabolic response of olive trees to the infection under controlled conditions, along with the evaluation of other types of biotic stresses known to occur under field conditions. This study aimed to evaluate the metabolic profile changes due to the infection with *X. fastidiosa* subsp*. pauca* ST53 in artificially inoculated young olive plants belonging to the variety *Cellina di Nardò,* one of the most susceptible cultivar under field conditions in the demarcated infected area of Apulia region. The test plants were grown in a thermo-conditioned greenhouse and were co-inoculated with the xylem-inhabiting fungi *Phaeoacremonium* and *Pseudophaemoniella* to partially reproduce the field conditions in terms of biotic stresses. Indeed, these xylem-inhabiting fungi were initially thought to have a role in OQDS^33–35^. The investigations were performed by combining NMR spectroscopy and MS spectrometry under an unbiased non-targeted approach for the analysis of leaf extracts and by applying multivariate statistical analysis to gain relevant information on the metabolites from the spectra.

## Material and methods

### Preparation of the olive plants

Two years-old olive plants of the cultivar Cellina di Nardò were used in the study. Plants were grown in a quarantine greenhouse under controlled conditions at a temperature of 24 ± 2 °C and a relative humidity over 80% at the CNR of Bari (Italy).

In order to simulate the field conditions, single and mixed inoculations were performed using the strain *X. fastidiosa* subspecies *pauca* ST53 in combination with the following fungal isolates obtained previously from dark-streaked olive sapwood (Table 1): *Phaeoacremonium aleophilum* B1a (F1), *Ph. rubrigenum* N20 (F2), *Pseudophaeomoniella oleae* Fv84 (F3), *Ps*. *oleicola* M24 (F4) or *Ps. oleicola* M51 (F5)^34, 35^.

**Table 1:** Features of the olive plants under investigation.

*X. fastidiosa* non-inoculated plants (Xf−)			*X. fastidiosa* inoculated plants (Xf+)		
	N. plants	Samples		N. plants	Samples
Control	3	6	Xf	4	10
F1	3	5	Xf-F1	4	6
F2	3	6	Xf-F2	3	8
F3	3	6	Xf-F3	3	6
F4	3	7	Xf-F4	3	6
F5	3	7	Xf-F5	3	5
Total	18	37	Total	20	41

Forty-eight plants were inoculated with a culture of *X*. *fastidiosa* ST53 using pinprick inoculation method as reported in the EPPO PM7/24 (3)^36^. A drop of inoculum (10–50 µL of the bacterial suspension) was placed at 3 consecutive leaf nodes on twigs located at 40–50 cm height on the trunk, and the plant tissue was pricked through the droplets, 5–6 times with a sterile entomological needle. Five to six shoots per plant were inoculated. The bacterial suspension was prepared by scraping and re-suspending in PSB buffer an 8–10 days old culture grown on BCYE solid medium. Bacterial suspensions were standardized to an OD600 value of 0.5–0.6, corresponding to an estimated cell concentration of 10^9^ CFU/mL. Control plants were inoculated in the same manner, using PSB buffer alone instead of the bacterial suspension. The bacterial colonization of the inoculated plants was periodically monitored by PCR^2,37^ following the test by Harper et al.^37^ recommended by EPPO PM 7/24 (3)^36^.

One month after the bacterial inoculation, the five fungal isolates were inoculated on the main trunk of 20 plants (4 per each fungal isolate), at 40–50 cm height from the soil level. The inoculation was performed by removing the bark with a sterile cork-borer (5 mm in diameter) and placing a mycelial plug (4 mm in diameter) taken from the edge of 14-day-old cultures of each isolate grown on Potato Dextrose Agar (PDA). Sterile, non-colonized PDA plugs were used to inoculate 4 control plants. The inoculation sites were then wrapped with sterile wet cotton and Parafilm to avoid dehydration. In order to get evidence of fungal colonization in the trunk, destructive tests were executed on some of inoculated replicates after 1.5 year post inoculation. Tests allowed to assess wood discoloration and fungal colonization at different distances from the inoculation point^34^. To this purpose, portions of the trunk were surface disinfected by dipping in a 3% aqueous solution of sodium hypochlorite (3 min), and then rinsed in sterile distilled water. The bark was removed aseptically, and portions (5 × 5 mm) of the wood were excised and plated onto PDA amended with streptomycin sulphate (300 mg/L). After 12–15 days incubation at 21 ± 2 °C, the development of colonies from the wood chips was determined. The occurrence of wood discolorations and the re-isolation of the target fungal isolates up to 20–40 cm from the inoculation point, confirmed the effective colonization of the plants by all the fungal isolates.

Among the inoculated plants, 20 plants systemically infected by *X. fastidiosa* and showing initial unambiguous symptoms of shoot dieback and desiccation were selected for sampling leaf tissues and subsequent laboratory analyses. However, only asymptomatic leaves from these symptomatic plants were sampled for laboratory metabolomics analyses.

Summarizing, the set of experimental plants included 18 mock-inoculated plants (bacterium-free control plants) and 20 *Xylella*-positive (Xf+) plants as summarized in Table 1.

Leaf sampling from inoculated (Xf+) and non-inoculated (Xf−) plants was performed 2 years after inoculations. Each sample was constituted by a statistically representative number of mature leaves (10–15 leaves corresponding to ca. 2 g) collected from the lower, middle and upper part of the plant. All the harvested samples were coded and stored in plastic bags at − 20 °C until analysis. After leaf sampling, all Xf+ plants were submitted to destructive tests to confirm the fungal colonization in the trunks.

The procedures for sample preparation, NMR and HPLC–MS spectra acquisition and multivariate statistical treatment of the spectroscopic data are detailed in Supplementary Information.

### Laboratory analysis

#### Reagents

All chemicals were of analytical reagent grade. The hydrochloric acid (37%), sodium oxalate (≥ 99.5%), deuterium oxide (99%D) and sodium azide (≥ 99.0%) were purchased from Sigma-Aldrich (Milan, Italy), while sodium salt of (trimethylsilyl)-propionic-2,2,3,3-d4 acid (TSP, 99%D) was purchased from Armar Chemicals (Döttingen, Switzerland). Acetonitrile and methanol LC/MS grade and isopropanol HPLC grade were purchased from Sigma-Aldrich (Milan, Italy). Water was doubly deionised (resistivity: 18 MΩ cm) with a Milli-Q water purification system (Merck Millipore, Darmstadt, Germany).

#### Samples preparation for NMR and HPLC–MS analysis

Olive leaves were freeze-dried at − 50 °C and 0.045 atm for 24 h in a lyophilizer (Martin-Christ GmbH, Model Alpha 1–4 LSC). Dried leaves were ground manually employing a mortar and pestle that were cleaned after each sample with absolute ethanol and an aspirator. The obtained powder was sieved (through a metallic sifter, pore size of 0.5 mm) and stored at room temperature under vacuum in a plastic bag protected from light, until analysis.

#### NMR measurements^38^

From each sample, 50 mg of olive leaf powder were placed into a test tube. The sample solution was prepared by adding 1.5 mL of oxalate buffer at pH 4.2 (pH value was reached after addition of 37% HCl to 100 mL an aqueous solution containing 0.25 M of Na_2_C_2_O_4_ and 2.5·10^–3^ M of NaN_3_), then sonicated for 10 min at 40 kHz, shaken for 5 min in a VORTEX at 2500 rpm, and centrifuged for 15 min at 4700 g. Then, 630 μL of the supernatant solution was transferred into an NMR tube, to which was added 70 μL of 0.20% of sodium salt of 3-trimethylsilyl-2,2,3,3-tetradeuteropropionic acid (TSP) solution in D_2_O.

One-dimensional ^1^H NOESY spectra were recorded on a Bruker Avance I 400 MHz spectrometer equipped with a 5 mm inverse probe and with an autosampler. ^1^H NOESY spectra were collected with 128 scans of 64 K data points with a spectral width of 8013 Hz, a pulse angle of 90°, an acquisition time of 4.09 s, a mixing time of 10 ms and a recycle delay of 3.0 s. Each spectrum was acquired using TOPSPIN 3.0 software (Bruker BioSpin GmbH, Rheinstetten, Germany) under an automatic process that lasted around 22 min and encompassed sample loading, temperature stabilization for 5 min, tuning, matching, shimming and 90° pulse calibration. Free induction decays (FIDs) were Fourier transformed, the phase was manually corrected, the baseline was automatically corrected, and the spectra were aligned by setting the TSP singlet to 0 ppm.

#### HPLC/MS measurements

The polyphenolic fraction present in olive leaf was extracted using methanol. Particularly, 10 mg of olive leaf powder were added to 3.0 mL of methanol, then mixed for 20 min through sonication, and lastly the supernatant was separated by centrifugation at 4700 g. The methanolic extract was filtered at 0.45 μm and was diluted 1:10 with methanol.

HPLC/ESI–MS analyses of olive leaf extracts were carried out in negative mode by a MicroTOF-Q II mass spectrometer (Bruker Daltonics, Macerata, Italy) in the range 50–1000 m/z, equipped with an ESI ion source with nitrogen as nebulizing gas (4 atm) and drying gas (10 L/min, 180 °C); capillary voltage at 3500 V and end plate offset at − 500 V.

Mass accuracy was verified by infusing a solution of Na-formate made up of 10 mL of 98% formic acid, 10 μL of sodium hydroxide (1.0 M), 490 μL of *i*-propanol and 490 μL of deionized water. Extracts were introduced (20 μL) in the HPLC (Agilent 1200 Series), using an autosampler in a RP column Synergi 4 µm Fusion-RP 80 Å, 100 mm × 3.0 mm. Flow rate was set to 0.5 mL/min using deionized water (resistivity: 18 MΩ cm, eluent A) and acetonitrile (LC/MS grade, eluent B) with 0.1% of formic acid, in gradient: 1% of B in the first minute and shifting to 100% B within the following 15 min. The composition was held 2 min, returning to 1% B in 2 min and keeping this condition for additional 3 min to achieve the column stabilization before next run (total run time was 18 min). The raw data were collected as continuum mass spectra at regular time interval (spectra rate of 1 spectrum/s); mass spectra were processed using Data Analysis 4.0 (Bruker Daltonik GmbH, Bremen, Germany).

#### Data analysis

The FIDs, relative to the 1D ^1^H NOESY NMR experiments carried out on 78 aqueous extracts of olive leaves, were processed by using Topspin 3.0 software (Bruker BioSpin GmbH, Rheinstetten, Germany), and segmented into 475 regular intervals (0.02 ppm-sized buckets) in the range of [10, 0.50] ppm by using AMIX 3.9.13 software (Bruker BioSpin GmbH, Rheinstetten, Germany). The underlying area of each bucket was normalized to the total intensity; the areas of the buckets in the region [5.10, 4.60] ppm, corresponding to the residual water signal, were set to 0.

Analogously, the respective 78 chromatograms produced by HPLC/ESI–MS were processed and stored in netCDF format by using Data Analysis 4.0 (Bruker Daltonik GmbH, Bremen, Germany), and then subjected to the advanced bucketing by using AMIX 3.9.13. This method of bucketing is internally based on picked peaks, and has two major advantages: (1) peaks existing in only one spectrum are stored in columns which have only zero entries otherwise, thus their significance is raised; (2) it allows a very fine bucketing, e.g. the spectrometers mass resolution may be taken as the bucket size. The advanced bucketing only creates columns if needed, thus reducing the table sizes in respect to a normal rectangular bucketing. The bucket regions were given in units of m/z, in the mass range [50, 750] m/z, with a delta mass of 0.01 m/z, from 0.60 min to 16.50 min of retention time. A noise level was selected as absolute counts (“min threshold” = 1000, “max threshold” = 700,000); spikes coming from electronic instabilities and isotopic peaks were removed in order to include only relevant information. The resulting table consisted of 889 buckets.

Both data matrices were imported into SIMCA 13.0.3 program (Umetrics, Umea, Sweden) to carry out multivariate statistical analyses (MVA). MVA reduces the dimensionality of large datasets by providing new variables, named latent or principal components (PCs), describing a model. The new coordinates of the observations are called scores, the weight of the original variables on each PC are called loadings. In the present study, the NMR spectra and the MS chromatograms constituted the observations, the buckets constituted the *x-*variables. Buckets were centred and subjected to Pareto scaling (each *x*_*j*_-variable was scaled to 1/sqrt(sd_*j*_), where sd_*j*_ is the standard deviation of *x*_*j*_-variable computed around the mean), giving each *x-*variable a variance equal to its own standard deviation and, thus avoiding noise inflation. Pareto scaling was considered the optimal scaling method for “omics” datasets with large dynamic ranges. First, an unsupervised MVA method, the Principal Component Analysis (PCA), was applied to get an overview of data. Then, a supervised approach, the Orthogonal Partial Least Square-Discriminant Analysis (OPLS-DA), was performed to identify those *x-*variables that discriminate between observations belonging to different classes a priori defined [in the present case, n. 41 samples infected by *X. fastidiosa* (Xf+) *versus* n. 37 samples not infected by *X. fastidiosa* (Xf−)].

## Results and discussion

### Data analysis

The non-targeted metabolomics approach allows the visualization of changes of metabolite composition of a sample. It provides suitable information to focus the specific molecules or groups of molecules involved in the phenomenon under investigation. In the present study, metabolomics datasets were acquired by both MS spectrometry and NMR spectroscopy, two complementary techniques that give mass and structural details of the molecules. The spectral features related to the infection-induced metabolite changes were extracted by using multivariate statistical analysis applied to both techniques and were used to identify metabolites which were known and unexpected. While MS spectra contained mass details in the range 50–750 m/z and were taken into account as a whole, the ^1^H-NMR spectra were considered excluding the region containing the residual solvent signal (for a typical ^1^H-NMR spectra see Supplementary Table S1 and Supplementary Fig. S1).

**Figure 1:**
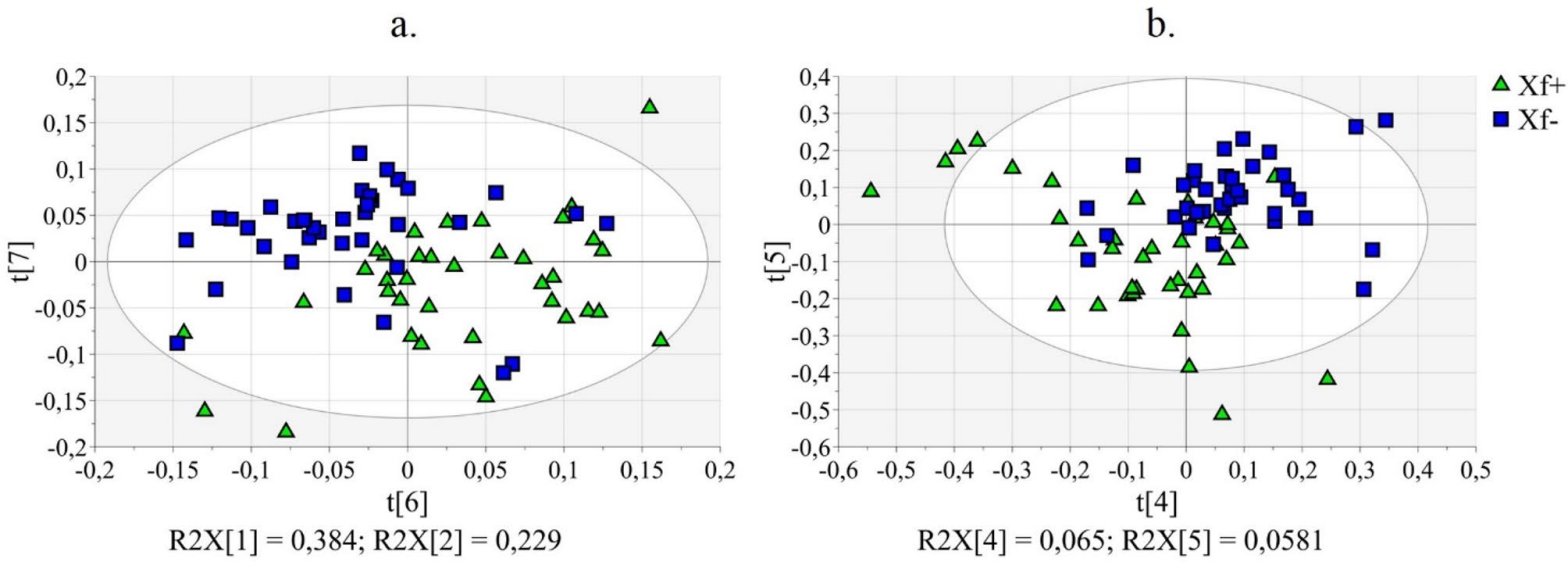
PCA applied to NMR and MS data obtained by analysis of olive leaves (Xf+: samples from *X. fastidiosa* inoculated plants; Xf−: samples from *X. fastidiosa* non-inoculated plants). (**a**) PCA applied to NMR data: t(6)/t(7) score plot relating to PC6/PC7; (**b**) PCA applied to MS data: t(4)/t(5) score plot relating to PC4/PC5.

Initially, Principal Component Analysis (PCA) was performed on each study group separately (Xf+ and Xf−) with the aim to assess the quality and homogeneity of the data. Quality of PCA models was evaluated based on the R^2^ (goodness-of-fit) and Q^2^ (goodness-of-prediction) parameters. By inspection of the Hotelling’s T^2^ plots, when NMR data were analysed, 4/78 outliers were identified in the Xf+ group and were removed from the dataset. PCA was carried out on 74 of the total 78 samples, indicating that about 7% of the *x*-variance (R^2^X[6] = 0.039 and R^2^X[7] = 0.030) was related to the *X. fastidiosa* infection of the plants (Fig. 1a). The outliers were 3/78 when MS data were considered and 13% of the *x*-variance was explained along PC4 and PC5 (R^2^X[4] = 0.065 and R^2^X[5] = 0.058, Fig. 1b). No significant effects of fungi co-infections on metabolic profile were recognized by PCA in these experiments. This finding suggests that the contribution of the fungi infections to total variance seems to be negligible.

Subsequently, Orthogonal Partial Least Square-Discriminant Analysis (OPLS-DA) was applied with the aim to find metabolites able to discriminate each category of samples (Xf+ vs. Xf−). In fact, OPLS-DA separates the variation of *x*-variables into two parts, the predictive part, correlated to the sample class, and the orthogonal part, uncorrelated to the class. As a result, model interpretability and identification of discriminating *x*-variables are improved. For OPLS-DA, quality of the models was evaluated taking into account the parameters R^2^Y (fraction of *y*-variance explained by the model), Q^2^, permutation tests and CV-ANOVA tests, avoiding data overfitting. In the present case, with two possible classes (Xf+ vs. Xf−), models presented only one predictive component and all other components reflected the orthogonal variation.

**Figure 2:**
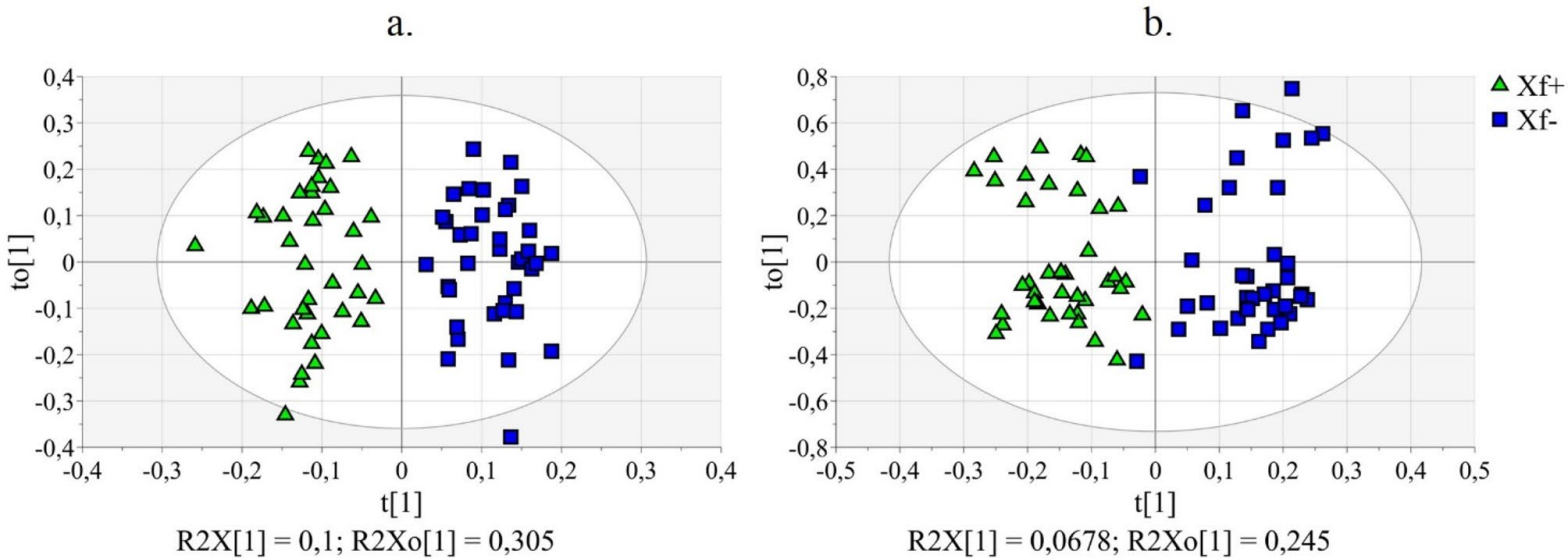
OPLS-DA applied to NMR and MS data obtained by the analysis of olive leaves (Xf+: samples from *X. fastidiosa* inoculated plants; Xf−: samples from *X. fastidiosa* non-inoculated plants). (**a**) OPLS-DA applied to NMR data: t(1)/t_O_(1) score plot relating to P1/O1; (**b**) OPLS-DA applied to MS data: t(1)/t_O_(1) score plot relating to P1/O1.

Considering NMR data, a 1 + 5 + 0 OPLS-DA model was obtained with one predictive component (P1) and 5 orthogonal components (O1–O5). P1 explained 10.0% of *x*-variance (R^2^X = 0.100) and modelled 87.2% of *y*-variance (R^2^Y = 0.872). Most part of *x*-variance (68.5%) was explained by O1–O5 (R^2^X[cum] = 0.685) (Supplementary Table S2). In Fig. 2a, P1 versus O1 (t[1] vs. t_O_[1]) scores plot shows the distribution of the observations which were much scattered along O1; in fact, O1 explained alone 30.5% of systematic information in *x*-space, orthogonal to *y*-space (R^2^X[O1] = 0.305). The robustness of the OPLS-DA model was ascertained by validation with a 200-permutation test (*y*-intercept for R^2^ was < 0.4 and for Q^2^ was < − 0.05 for both classes, Supplementary Fig. S2) and with CV-ANOVA test (the computed p-value was 2.06·10^–13^, Supplementary Table S2).

Considering MS data, a 1 + 2 + 0 OPLS-DA model was obtained with the predictive component P1 explaining 6.8% of *x*-variance (R^2^X = 0.068) and modelling 83.1% of *y*-variance (R^2^Y = 0.831) (Fig. 2b; Supplementary Table S3). The two orthogonal components, O1 and O2, explained 35.4% of the *x*-variance (R^2^X[cum] = 0.354) (Supplementary Table S2). Also for MS data, the robustness of the OPLS-DA model was ascertained by validation with a 200-permutation test (*y*-intercept for R^2^ was < 0.4 and for Q^2^ was < − 0.05 for both classes, Supplementary Fig. S3) and with CV-ANOVA test (the p-value computed was 3.56 × 10^–18^, Supplementary Table S3).

**Figure 3:**
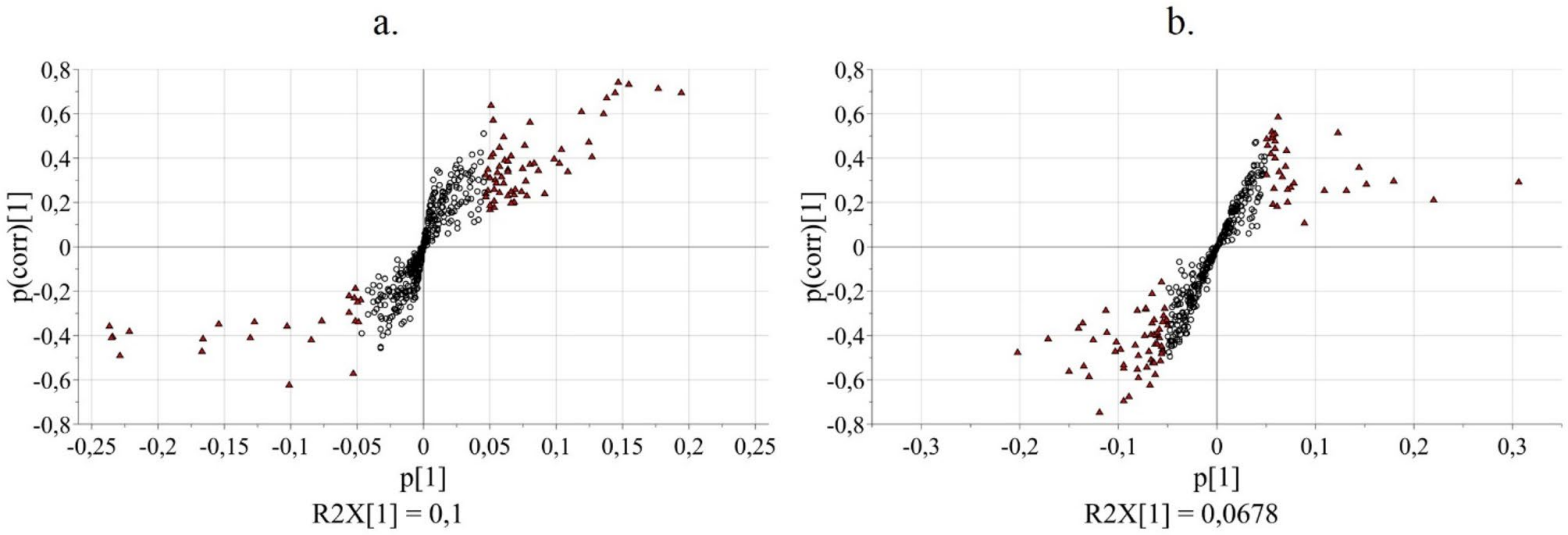
Identification of metabolites with the highest potential as biomarkers by means of the S-plots obtained by applying OPLS-DA to NMR data (**a**) and MS data (**b**). NMR and MS data derives from the analysis of olive leaf samples. The values of p(1) and pcorr(1), relating to the predictive component P1, are displayed; red triangles correspond to x-variables having VIP_predictive_ value > 1.

In order to identify the metabolites that were differently expressed between infected (Xf+) and non-infected (Xf−) samples, VIP (Variable Importance in the Projection) predictive values (Tables 2 and 3) and S-plot (Fig. 3) were examined. VIP values indicate the importance of each *x*-variable on the predictive part of the model and are reported in Tables 2 and 3. Values larger than 1 are the most relevant for explaining the *y*-response. The S-plot displays the p[1] versus p(corr)[1] vectors of the predictive component, where p[1] is the loading vector that expresses the weight of each *x*-variable on the selected component P1, and p(corr)[1] is p[1] scaled as a correlation coefficient between each *x*-variable and t[1], ranking from − 1.0 to 1.0. In the S-plot, the *x*-variables situated far out on the wings of the S combine high model influence with high reliability and are relevant in the search for up- or down-regulated markers. The variables related to metabolites with the highest potential as biomarkers were selected among those with VIP_predictive_ > 1.0 and |p(corr)|> 0.5 (Tables 2 and 3)^39^.

**Table 2:** Selected NMR variables related to metabolites with the highest potential as biomarkers (VIP_predictive_ > 1.0 and |p(corr)|> 0.5). NMR data derives from the analysis of olive leaf samples.

NMR bucket	VIP_predictive_	p[1]	p(corr)[1]
4.07	2.1365	− 0.1008	− 0.6235
2.17	1.1265	− 0.0531	− 0.5710
2.65	1.7060	0.0805	0.5618
2.63	1.1052	0.0521	0.5744
2.57	2.8698	0.1354	0.6024
2.51	2.5312	0.1194	0.6089
8.43	1.0775	0.0508	0.6400
2.53	2.9305	0.1382	0.6734
2.75	4.1134	0.1940	0.6935
2.55	3.0618	0.1444	0.6939
4.35	3.7571	0.1772	0.7152
4.37	3.2835	0.1549	0.7313
4.33	3.1085	0.1466	0.7413

**Table 3:** Selected MS variables related to metabolites with the highest potential as biomarkers (VIP_predictive_ > 1.0 and |p(corr)|> 0.5). MS data derives from the analysis of olive leaf samples.

MS bucket	VIPpred	p[1]	p(corr)[1]
487.3470	2.3961	− 0.1191	− 0.7475
491.1210	1.9123	− 0.0951	− 0.6940
299.0400	1.5957	− 0.0896	− 0.6740
695.4000	1.3664	− 0.0679	− 0.6216
299.0290	1.8027	− 0.0793	− 0.5894
623.1440	2.6166	− 0.1301	− 0.5835
379.0900	1.2551	− 0.0624	− 0.5767
609.1490	3.0210	− 0.1502	− 0.5632
469.3320	1.6273	− 0.0809	− 0.5494
215.0950	1.8982	− 0.0944	− 0.5452
615.2110	1.4210	− 0.0706	− 0.5440
491.1540	2.7100	− 0.1347	− 0.5355
539.1940	1.9076	− 0.0948	− 0.5332
603.0840	1.3106	− 0.0651	− 0.5231
715.2290	1.2820	− 0.0637	− 0.5226
577.2700	1.2920	− 0.0642	− 0.5171
331.0730	1.1573	− 0.0575	− 0.5156
439.0910	1.3476	− 0.0670	− 0.5080
607.3300	1.1494	0.0571	0.5075
405.1040	1.1761	0.0585	0.5126
133.0130	2.4626	0.1224	0.5166
115.0020	1.1206	0.0557	0.5185
511.1860	1.2433	0.0618	0.5851

### Markers identification

NMR and MS signals were attributed by comparison with either signal of authentic samples or a reference database and literature. In some cases, NMR signal attribution was supported by Chenomx 8.3 database and additional 2D-NMR experiments, while MS signal attribution was supported by HMDB (Human Metabolome Database). Moreover, NMR-MS correlation analysis resulted effective for identifying metabolites as it combined NMR and MS spectral data.

**Figure 4:**
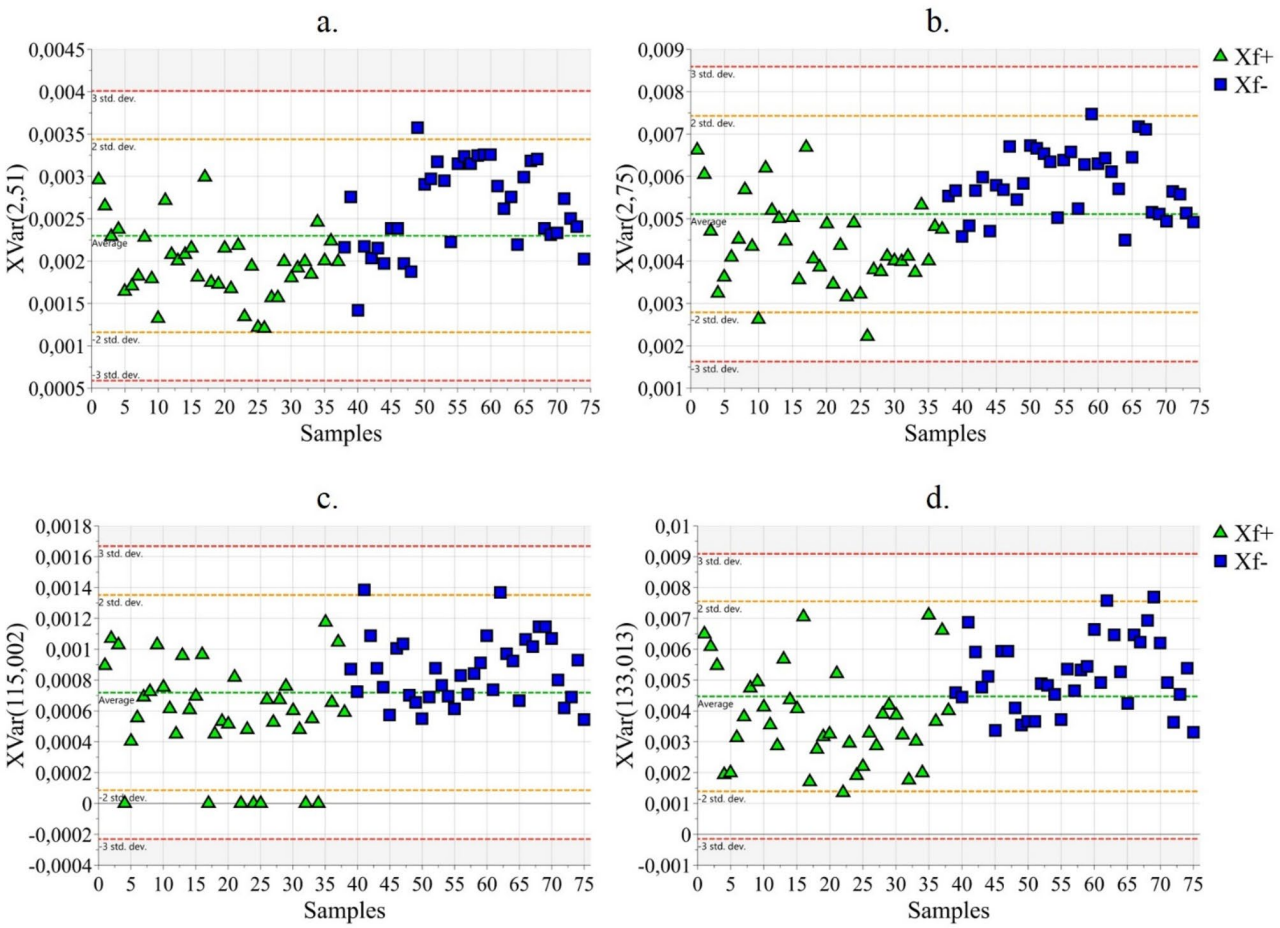
Statistic of the buckets attributable to malic acid [(**a**) 2.51 ppm; (**b**) 2.75 ppm; (**c**) 115.002 m/z; (**d**) 133.013 m/z]; Xf+: samples from *X. fastidiosa* inoculated plants; Xf−: samples from *X. fastidiosa* non-inoculated plants.

NMR data analysis revealed that the buckets centred at 2.51, 2.53, 2.55, 2.57, 2.63, 2.75, 4.33, 4.35, and 4.37 ppm are characterized by higher intensity in Xf− samples with respect to Xf+ ones. These buckets can be attributed to the signals of malic acid. It is important to notice that the chemical shifts of the malic acid are not strictly identical for all the samples due to their dependence on ion strength of the solution and then, on the specific features of the sample. Moreover, in the same regions, also signals of other molecules overlapped and could be responsible for the observed trend. In order to avoid misinterpretation, a correlation matrix was created by combining NMR and MS data. It resulted that the abovementioned NMR buckets correlated with two MS buckets corresponding to malic acid (monoisotopic molecular weight (MMW) = 134.0215). In particular, a correlation value of about 0.6 was found between the NMR buckets at 2.51 e 2.75 ppm and the MS buckets at 133.013 and 115.002 m/z, the latter being attributable to [M-H]^−^ and [M-H-H_2_O]^−^ fragments, respectively. In Fig. 4, the statistics of the NMR buckets at 2.51 e 2.75 ppm and of the MS buckets at 133.013 and 115.002 m/z are reported.

Malic acid is one of the most common organic acid in plant tissues. It is involved in important biosynthetic processes such as the synthesis of amino acids and the citric acid cycle^40^. Moreover, its anionic form is implicated in defence-related deposition of lignin and in microbial pathogens inactivation processes^41^. Thus, modifications in malate content may be attributed to stress conditions of the plants.

Also, formic acid showed the same trend as malic acid, i.e. higher intensity in infected samples, as ascertained by the statistic of the NMR bucket at 8.43 ppm (Supplementary Fig. S4).

The NMR bucket at 4.07 ppm resulted more intense in Xf+ leaves (Supplementary Fig. S5). It was assigned to sucrose after ascertaining that it correlates with MS buckets at 341.111 [M-H]^–^, 377.096 [M + Cl]^–^, 387.118 [M + HCOO]^ –^  and 683.229 m/z [2 M-H]^–^ with high correlation values ranging from 0.60 to 0.90. The same MS buckets correlated also with NMR buckets at 5.39 and 5.41 ppm. These buckets include the signal of the C^1^-*H* of the glucopyranosyl ring of the sucrose. The distribution of sucrose was evaluated along with other polyols. In fact, expanding the analysis to NMR buckets with 0.3 <|p(corr)|< 0.5 it was found that most buckets in the range 3.90–3.64 ppm followed a similar trend to that of sucrose. Especially, the buckets at 3.65, 3.69, 3.75, 3.77, 3.79, 3.81, 3.85, and 3.89 ppm (Supplementary Fig. S6) correlated with the MS buckets at 181.073 [M-H]^–^, 163.061 [M-H-H_2_O]^–^, and 363.149 m/z [2 M-H]^–^ (correlation values ranged from 0.60 to 0.87), thus they were ascribed to mannitol (MMW = 182.0790). Mannitol is a sugar alcohol product of the primary photosynthetic metabolism in mature leaves. It represents one of the major carbohydrates which is translocated to plant tissues through the phloem^42–44^. Mannitol plays many roles in plant growth as well as in plant protection being a carbon and energy source^42^, but also an osmoprotectant against drought^45,46^, salinity^47,48^ and oxidative stress^49^. Mannitol is involved also in plant-pathogen interactions. Some fungi produce mannitol to protect themselves against plant-defence mechanisms^50–58^. It has been reported that, in plant-pathogen interactions, mannitol acts as a quencher of reactive oxygen species (ROS)^52^. ROS production has been reported to occur more importantly in *X. fastidiosa* infected olive trees compared to the *Xylella*-negative ones^26^. Also, the activities of some antioxidant enzymes related to ROS-scavenging activity have been found increased in *X. fastidiosa*-infected olive plants^58^. In the present case, mannitol amount was found higher in all *X. fastidiosa*-infected plants with respect to the non-infected ones regardless their biotic conditions (fungal infections). As a deduction, mannitol was probably produced in response to the *X. fastidiosa* infection, so to balance cell reinforcements against ROS induced by *Xylella*.

An opposite trend was shown by buckets centred at 5.77, 6.01, 6.03, and 6.05 ppm, whose intensities were found (on average) higher in Xf− leaf samples with respect to Xf+ ones (Supplementary Fig. S7). Such buckets, strictly correlated with the bucket 2.65 (Table 2), correlated also with the MS bucket at 539.181 [M-H]^–^ (correlation value of 0.75), thus they were assigned to oleuropein (MMW = 540.1843). Moreover, a correlation was found also with the NMR buckets at 1.57, 1.59, 2.49, 5.75, and 7.51 ppm (correlation values ranging from 0.92 to 0.99), where the other signals of oleuropein are contained. Oleuropein is a chemical compound found in olive leaves, whose antioxidant properties are well-known. The fact that this kind of compounds resulted to be less abundant in leaves of infected plants (Xf+) supported their biological role of sacrificial molecules even though an opposite trend was reported for the aldehydic form of oleuropein in Ogliarola Salentina leaf samples^28^.

Finally, two potential markers of the infection could not be fully characterised by the available data but deserve mentioning. The evidence for such markers comes from the NMR bucket at 2.17 ppm and the MS bucket at m/z 487.347. Concerning the NMR bucket at 2.17 ppm, higher intensities were found in infected samples (Supplementary Fig. S8). It was tentatively assigned to acetoin which is an important physiological metabolite excreted by many microorganisms^59^. Also, the intensities of the MS bucket at m/z 487.347 were found higher for infected samples (Supplementary Fig. S9). Attention should be paid to these findings with the aim to unequivocally disclose the identity of the molecules generating such signals.

## Conclusions

In this work, a combined non-targeted NMR/MS study was carried out to identify the metabolome changes in young *Cellina di Nardò* plants due to the *X. fastidiosa* infection under controlled greenhouse conditions, besides the co-infections of some xylem-inhabiting fungi that were made on the same plants, to partly reproduce the field environment in terms of biotic stresses. The combined approach involving NMR, HPLC-HRMS and multivariate statistical analysis showed that changes of the amounts of malic acid, formic acid, mannitol, sucrose and oleuropein were caused exclusively by *X. fastidiosa,* regardless of the co-infections with fungi*.* In particular, identification of mannitol as a discriminant metabolite in the case of *X. fastidiosa* infection in olive trees paralleled what was reported for citrus plants infected by Huanglongbing disease^60^. Among the observed changes, those of malic acid and oleuropein have been reported in previous studies on *X. fastidiosa* infections in olive groves, thus confirming that such a pathogen affects both primary and secondary metabolism in the olive trees. All these findings suggest that further studies should follow this first trial conducted under controlled conditions with the aim to gain deeper insights on the role of primary and secondary metabolites at the level of olive cultivars tolerance to *X. fastidiosa* infections.

## Supplementary Information

Supplementary Information.
