# Supplementary material for: A non-targeted metabolomics study on Xylella fastidiosa infected olive plants grown under controlled conditions
Source: Sci Rep. 2021 Jan 13;11:1070. doi: 10.1038/s41598-020-80090-x (PMC7806896; doi:10.1038/s41598-020-80090-x)
Supplement: Supplementary file 1 — Supplementary Information. [file 41598_2020_80090_MOESM1_ESM.docx]

**Supplementary Information**

**A non-targeted metabolomics study on *Xylella* *fastidiosa* infected olive plants grown under controlled conditions**

Asmae Jlilat^1^, Rosa Ragone^2,3^, Stefania Gualano^4^, Franco Santoro^4^, Vito Gallo^2,3,^*, Leonardo Varvaro^1^, Piero Mastrorilli^2,3^, Maria Saponari^5^, Franco Nigro^6^ and Anna Maria D’Onghia^4^

^1^Dipartimento di Scienze Agrarie e Forestali (DAFNE), Università degli Studi della Tuscia, via San Camillo de Lellis, 01100 Viterbo, Italy.

^2^Dipartimento di Ingegneria Civile, Ambientale, del Territorio, Edile e di Chimica (DICATECh), Politecnico di Bari, via Orabona 4, I-70125 Bari, Italy.

^3^Innovative Solutions S.r.l. – Spin Off del Politecnico di Bari, zona H 150/B, I-70015 Noci (BA), Italy.

^4^Centre International de Hautes Etudes Agronomiques Méditerranéennes (CIHEAM) of Bari, Via Ceglie 9, Valenzano (BA) 70010, Italy.

^5^Istituto per la Protezione Sostenibile delle Piante, CNR, SS Bari, Via Amendola 165/A, 70126 Bari, Italy.

^6^Dipartimento di Scienze del Suolo, della Pianta e degli Alimenti, Università degli Studi Aldo Moro, Via Amendola 165/A, 70126 Bari, Italy.


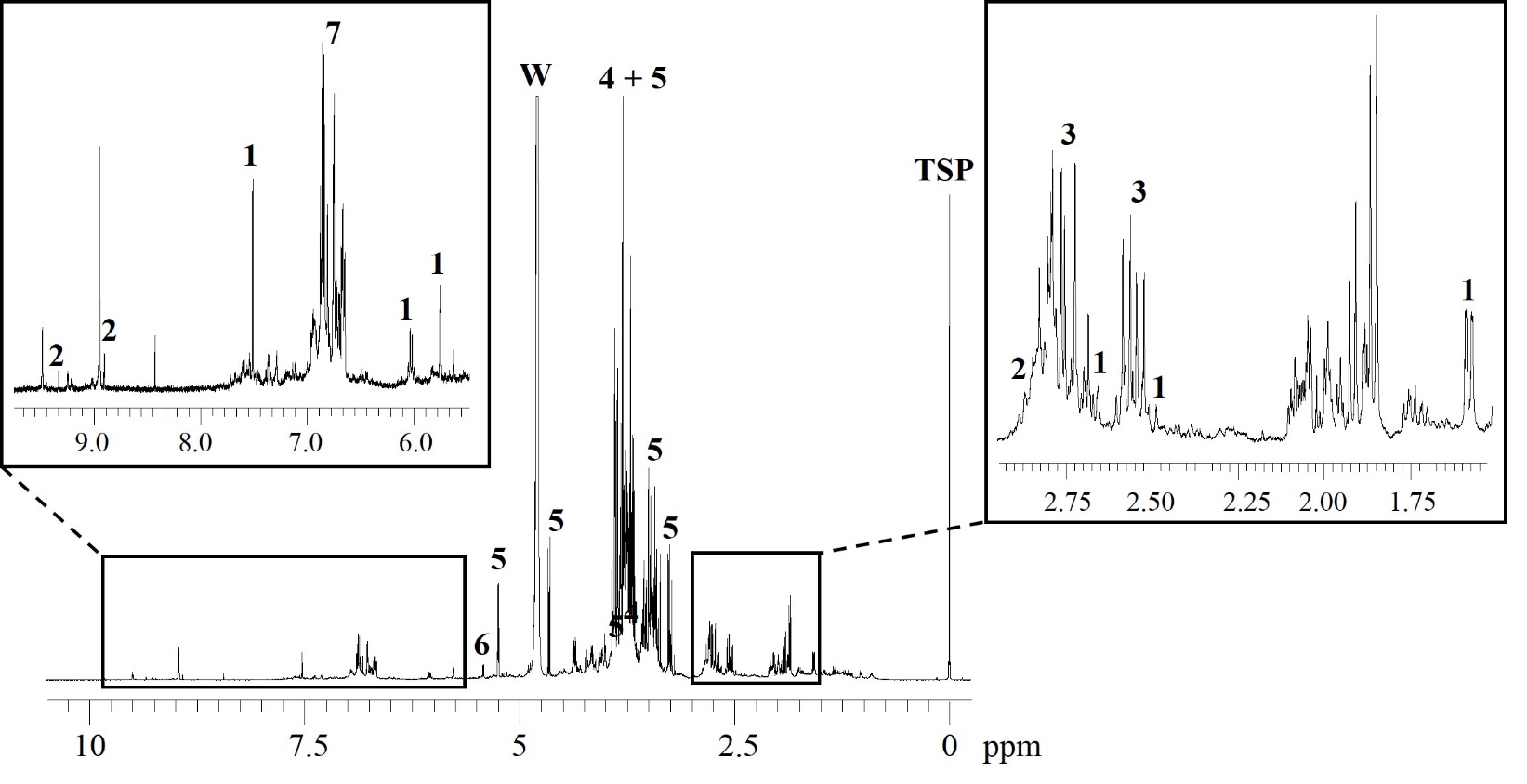


**Supplementary Figure S1.** ^1^H NOESY NMR spectrum of a water extract obtained from an olive leaf sample. Peak numbering is reported in first column of Table Supplementary Table S.1; “W” refers to the residual water signal.

**Supplementary** **Table S.1.** Assignment of relevant metabolites in the ^1^H NMR spectrum of *X. fastidiosa* infected leaves.

| Signal | Metabolite | δ (ppm) | MMW | Correlated  MS bucket [M-H]^-^ |
| --- | --- | --- | --- | --- |
| 1 | Oleuropein | 1.57, 2.49, 2.65, 5.76, 6.03, 7.51 | 540.184 | 539.182 |
| 2 | Oleuropein aglycone | 2.85 – 2.89, 8.91, 9.33 | 378.132 | 377.118 |
| 3 | Malic acid | 2.54, 2.75, 4.45 | 134.022 | 133.013 |
| 4 | Mannitol | 3.66 – 3.90 | 182.079 | 181.073 |
| 5 | Glucose | 3.25, 3.38 – 3.56, 3.65 – 3.90,  4.65 (anomeric-H β-glucose),  5.24 (anomeric-H α-glucose) | 180.063 | 179.057 |
| 6 | Sucrose | 5.40, 4.21, 4.06 | 342.116 | 341.111 |
| 7 | Aromatic compounds | 6.80 – 7.00 | - | - |

**Supplementary Table S2.** Parameters describing the OPLS-DA model built on NMR data, and CV-ANOVA test results.

| **Component** | **R^2^X** | **R^2^X(cum)** | **R^2^** | **R^2^(cum)** | **Q^2^** | **Q^2^(cum)** |
| --- | --- | --- | --- | --- | --- | --- |
| **Model** |  | **0.785** |  | **0.872** |  | **0.735** |
|  |  |  |  |  |  |  |
| **Predictive** |  | **0.100** |  | **0.872** |  | 0.735 |
| P1 | 0.100 | 0.100 | 0.872 | 0.872 | 0.735 | 0.735 |
|  |  |  |  |  |  |  |
| **Orthogonal in X (OPLS)** |  | **0.685** |  | **0.000** |  |  |
| O1 | 0.305 | 0.305 | 0.000 | 0.000 |  |  |
| O2 | 0.120 | 0.425 | 0.000 | 0.000 |  |  |
| O3 | 0.188 | 0.613 | 0.000 | 0.000 |  |  |
| O4 | 0.034 | 0.646 | 0.000 | 0.000 |  |  |
| O5 | 0.038 | 0.685 | 0.000 | 0.000 |  |  |
|  |  |  |  |  |  |  |
|  | **SS** | **DF** | **MS** | **F** | **p** | **SD** |
| **Total corr.** | 73.000 | 73.000 | 1.000 |  |  | 1.000 |
| **Regression** | 53.650 | 12.000 | 4.471 | 14.094 | **2.056·10^-13^** | 2.114 |
| **Residual** | 19.350 | 61.000 | 0.317 |  |  | 0.563 |


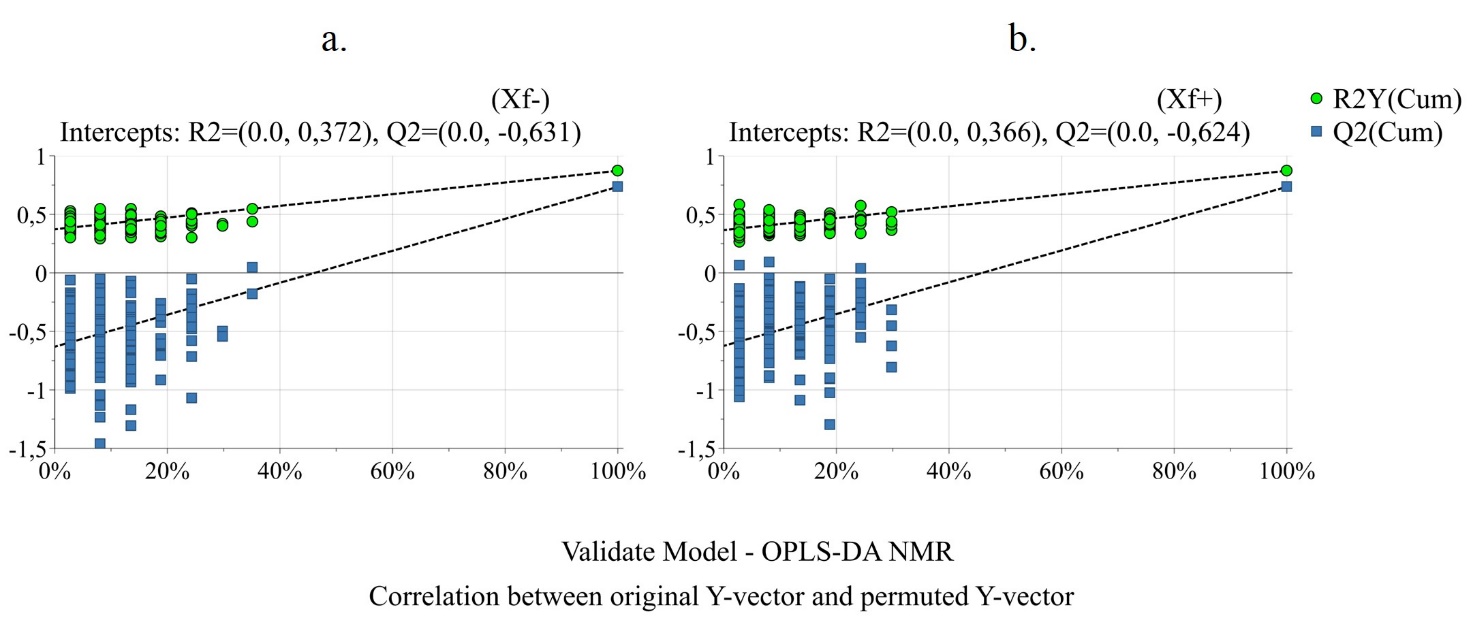


**Supplementary** **Figure S.2.** Permutation plots obtained for the OPLS-DA model built on NMR data: a 200-permutation test was applied for both classes (a: Xf-; b: Xf+).

**Supplementary Table S.3.** Parameters describing the OPLS-DA model built on MS data, and CV-ANOVA test results.

| Component | R^2^X | R^2^X(cum) | R^2^ | R^2^(cum) | Q^2^ | Q^2^(cum) |
| --- | --- | --- | --- | --- | --- | --- |
| Model |  | **0.422** |  | **0.831** |  | **0.742** |
| Predictive |  | **0.0678** |  | **0.831** |  | **0.742** |
| P1 | 0.0678 | 0.0678 | 0.831 | 0.831 | 0.742 | 0.742 |
| Orthogonal in X (OPLS) |  | **0.354** |  | **0** |  |  |
| O1 | 0.245 | 0.245 | 0 | 0 |  |  |
| O2 | 0.109 | 0.354 | 0 | 0 |  |  |
|  |  |  |  |  |  |  |
| CV-ANOVA test | **SS** | **DF** | **MS** | **F** | **p** | **SD** |
| Total corr. | 74.000 | 74.000 | 1.000 |  |  | 1.000 |
| Regression | 54.897 | 6.000 | 9.150 | 32.570 | **3.563·10^-18^** | 3.025 |
| Residual | 19.103 | 68.000 | 0.281 |  |  | 0.530 |

**
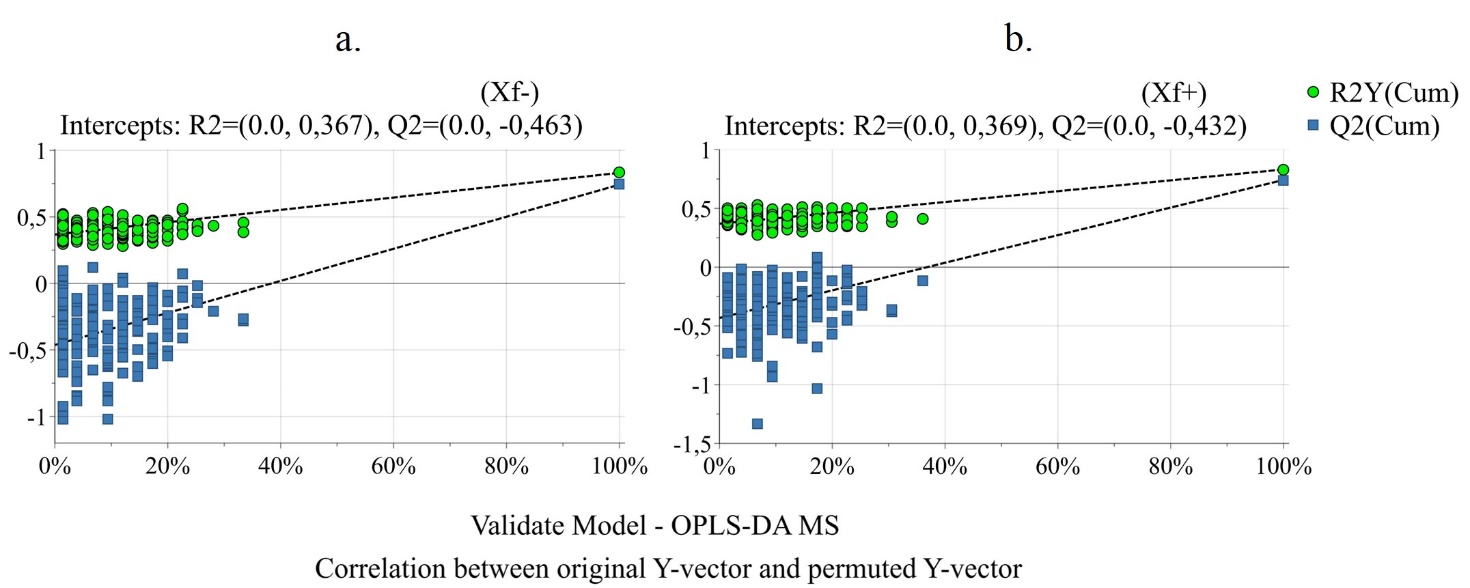
**

**Supplementary** **Figure S.3.** Permutation plots obtained for the OPLS-DA model built on MS data: a 200-permutation test was applied for both classes (a: Xf-; b: Xf+).

**
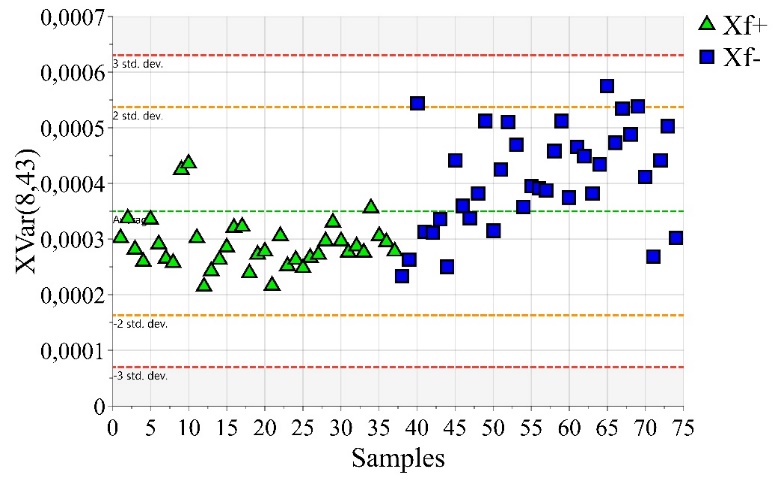
**

**Supplementary** **Figure S.4.** Statistic of the NMR bucket attributed to formic acid (8.43 ppm).


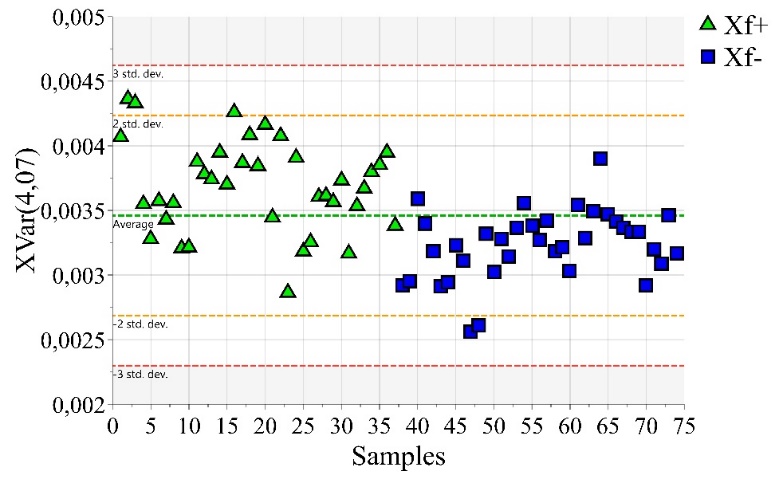


**Supplementary** **Figure S.5.** Statistic of the NMR bucket attributed to sucrose (4.07 ppm).


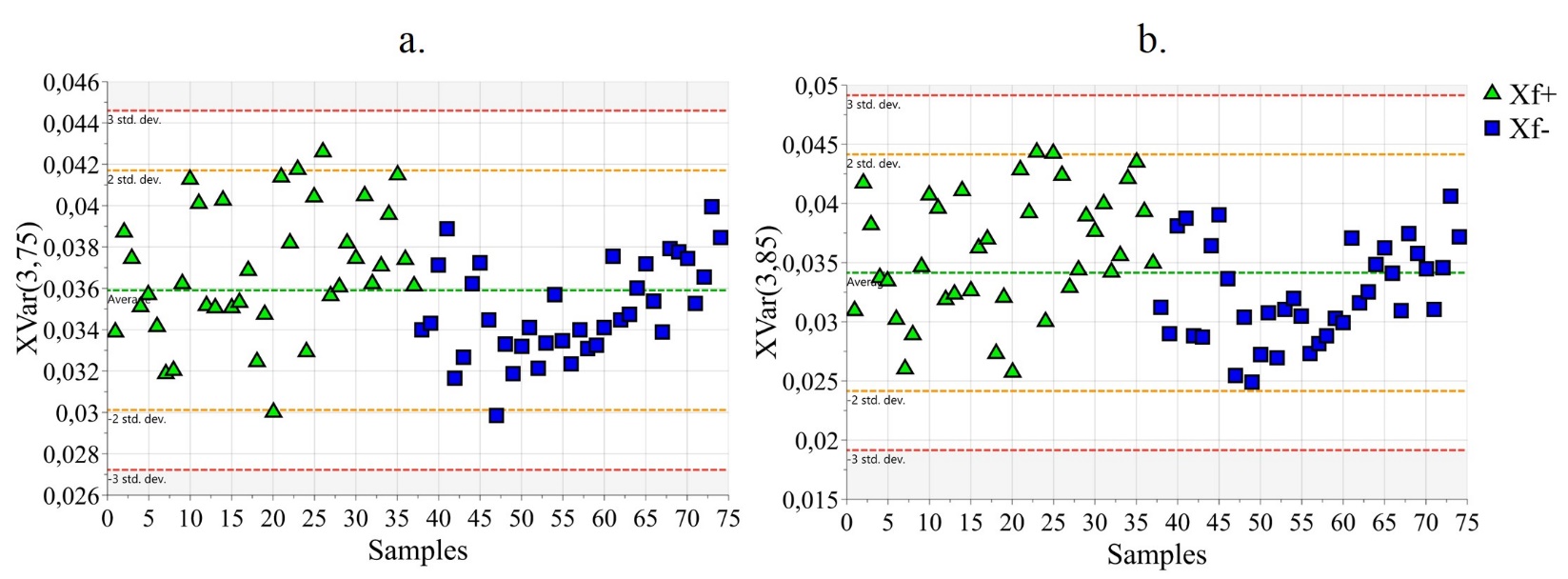


**Supplementary** **Figure S.6.** Statistic of the NMR buckets attributed to mannitol (a: 3.75 ppm; b: 3.85 ppm).


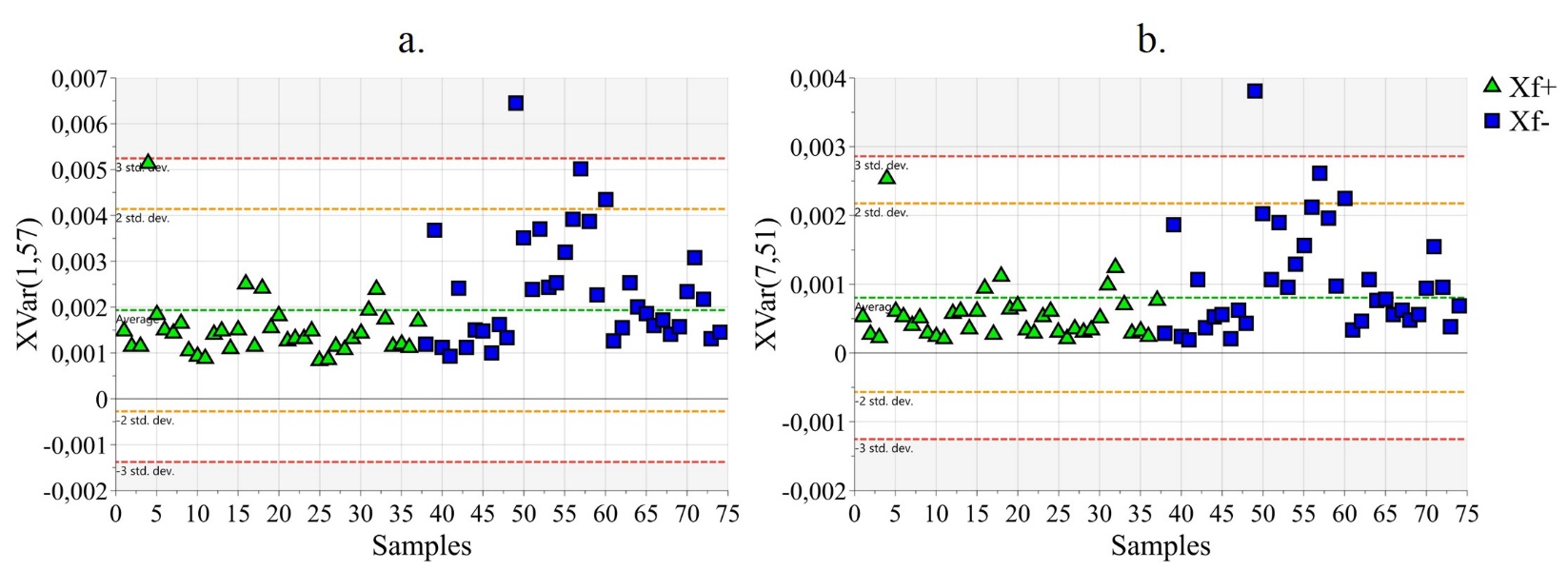


**Supplementary** **Figure S.7.** Statistic of the NMR buckets attributed to oleuropein (a: 1.57 ppm; b: 7.51 ppm).


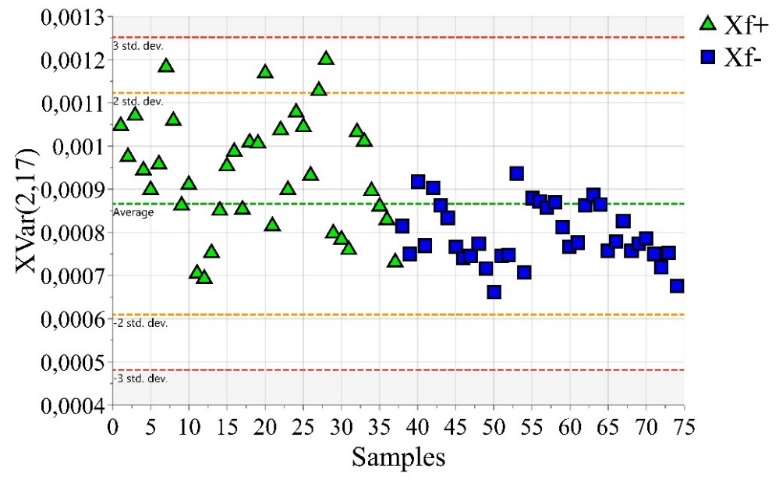


**Supplementary** **Figure S.8.** Statistic of the NMR bucket attributable to acetoin (2.17 ppm).


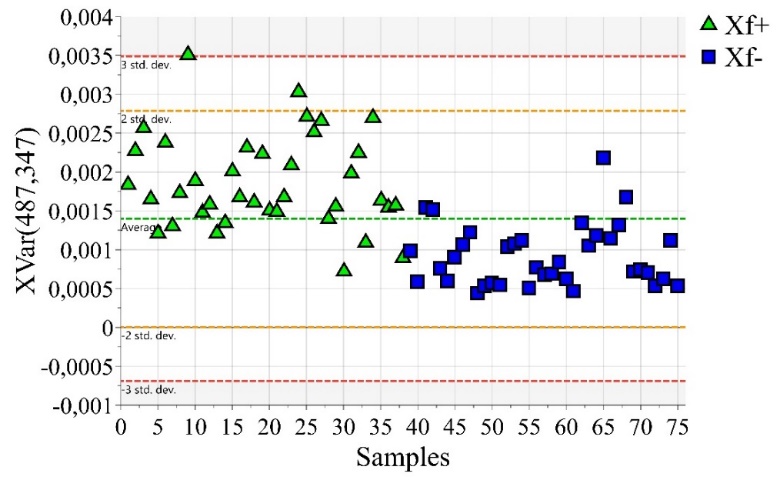


**Supplementary** **Figure S.9.** Statistic of MS bucket = 487.347 m/z, not assigned.
